# Important factors in municipality-based pediatric palliation from healthcare professionals’ perspective: A qualitative study

**DOI:** 10.1177/26323524241312915

**Published:** 2025-01-26

**Authors:** Kristin Skjærseth, Natalie Preminger, May Aasebø Hauken

**Affiliations:** Center for Crisis Psychology, Faculty of Psychology, University of Bergen, Bergen, Norway; Center for Crisis Psychology, Faculty of Psychology, University of Bergen, Bergen, Norway; Center for Crisis Psychology, Faculty of Psychology, University of Bergen, Postbox 7807, Bergen 5020, Norway

**Keywords:** healthcare professionals, home care, municipality healthcare, pediatric palliation, qualitative study

## Abstract

**Background:**

Municipality-based pediatric palliative care (PPC) is recommended to promote the quality of life for the child and family by enabling them to stay at home as much as possible. However, municipality-based PPC presents complex challenges and places significant demands on healthcare professionals. Yet, it remains an underexplored field.

**Objectives:**

Semi-structured and individual interviews with 16 healthcare professionals with experiences from PPC were conducted and transcribed. Data was analyzed using systematic text condensation.

**Results:**

To increase the knowledge base and understanding of important factors for municipality-based PPC from healthcare professionals’ perspective.

**Design:**

A qualitative method with an interpretive descriptive design was applied.

**Methods:**

The bridging theme “Establishing a sense of security and predictability for the family and healthcare professionals” emerged from the analysis. This was elaborated by three main themes: (1) “A comprehensive approach to the family,” (2) “Establishing and maintaining a dedicated, multidisciplinary pediatric palliative team in the municipality,” and (3) “Collaboration and communication between involved services.” Each main theme was further elaborated by subthemes.

**Conclusion:**

The importance of establishing security and predictability in municipality-based PPC was emphasized. To achieve this, holistic support for the entire family and ensuring sufficient competence in the municipality seem crucial. Establishing municipality PPC teams is proposed, and the need for early referral, routines for collaboration, and a designated coordinator appear to be key systemic factors.

**Registration and reporting guidelines:**

The study is registered in the institutional system for research project (RETTE ID: R2082), and the study is reported according to the COREQ checklist for qualitative studies.

## Plain language summary

Enhancing home-based care for children with life-limiting conditions

When children face serious health conditions, it’s often best for them to receive care at home, surrounded by family. This type of care, known as pediatric palliative care (PPC), focuses on improving quality of life for both the child and their loved ones. However, providing this care at a municipality level can be challenging and requires skilled healthcare professionals.

A study was conducted to better understand what makes municipality-based PPC effective from the viewpoint of these professionals. We interviewed 16 healthcare workers experienced in PPC, analyzing their insights to identify key factors for successful care.

The study found that creating a secure and predictable environment is essential for both families and healthcare workers. This involves a holistic approach that considers the family’s overall needs, forming a specialized team that can address the complex aspects of care, and ensuring clear communication between different services involved in the child’s care.

In conclusion, the study highlights the need for a well-coordinated team and support system within the municipality to provide effective PPC. This includes having a team dedicated to PPC, early involvement of care services, established routines for working together, and a coordinator to oversee the process. By focusing on these areas, municipality-based PPC can offer meaningful support to families during challenging times.

## Introduction

In Norway, it is estimated that about 8000 children suffer from a life-threatening or life-limiting condition that necessitates pediatric palliative care (PPC), of whom approximately 20% have cancer, 40% have a genetic condition, and 40% have a neuromuscular condition.^1^ This number is based on British estimates, which also predict an increase in the prevalence.^2^ The World Health Organization (WHO) defines PPC as the active and total care of the child’s body, mind, and spirit, including support to the family. PPC is not confined to end-of-life care; it should be initiated upon diagnosis of a life-limiting or life-threatening condition, encompassing a variety of diagnoses and trauma, from birth onwards.^2,3^ Thus, PPC adopts a holistic and family-oriented approach aiming to promote the quality of life and address the needs, choices, and wishes of children and their families.

PPC differs from adult palliation in several ways, which poses unique challenges. The heterogeneity of children requiring PPC, which includes a wide range of diagnoses and developmental stages, necessitates specific competencies. These include sensitive communication skills, the ability to manage complex symptoms, proficiency in clinical decision-making, and the capacity to support the entire family affected by the child’s condition.^4^ Consequently, PPC demands high levels of healthcare professional expertise and effective organization within primary and specialist healthcare systems. To optimize the family’s quality of life during palliative care, international standards for PPC state that healthcare services should provide municipality-based care.^5^ This aligns with guidelines in PPC from WHO and the United Nations Convention on the Rights of the Child.^4,6^

As in other countries, PPC is a relatively new field in Norway as the focus has primarily been on specialist healthcare services.^1^ However, aligning with international guidelines, the organization of palliative services for children in the municipalities is now mandated,^7^ and the government recommends establishing municipality-based PPC teams in primary healthcare.^8^ Additionally, each municipality in Norway must have a “child coordinator” to facilitate interdisciplinary collaboration across different service levels and providers.^9^ Healthcare professionals are also legally obliged to screen and follow-up siblings both during and after the course of illness.^10^

There is growing evidence that families prefer municipality-based care,^5,11–13^ as it enables families to stay home as much as possible, facilitating the most normal life possible for the child, siblings, and parents. This also enables the child to be allowed to die at home. Although municipality-based care can be provided by both specialist and primary healthcare services, most families in palliative treatment need various municipality services, including around-the-clock healthcare at the end of life.^13^ Municipality-based PPC is associated with improved child and parental quality of life,^14^ reduced symptom burden,^15,16^ and increased likelihood of home death.^16^ Despite this knowledge, the implementation of municipality-based PPC presents significant challenges. Children still receive more aggressive end-of-life treatment^17^ and are more likely than adults to die in hospital,^17–19^ which may be significant factors contributing to the challenges in achieving municipality-based PPC. Firstly, the rarity of families needing PPC in municipalities leads to limited exposure and maintenance of relevant knowledge and skills for healthcare professionals.^20^ Healthcare professionals in municipality settings also face emotional demands.^21,22^ Moreover, inadequate expertise^13,21,23^ and guidance^23^ place healthcare professionals at risk of increased stress levels.^13,21^ Collaboration between specialist and municipality services may also be lacking or difficult to manage.^23^ Proposed solutions to address these challenges include interdisciplinary teams,^13,20,24^ education,^13,23,24^ and comprehensive and early cooperation across services.^20,25^ The importance of building trustful relationships between healthcare professionals, the child, and the family has been emphasized.^12,14,23^ However, important factors, understood as the various elements or conditions that influence the provision of municipality-based PPC from the perspective of healthcare professionals, remain limited.

## Study aim

The overall aim of this study was to increase the knowledge base and understanding of important factors of municipality-based PPC from the perspective of healthcare professionals. More specifically, this study aims to answer the research question: Which factors do healthcare professionals experience as important for municipality-based pediatric palliation?

## Method

Based on the research question, a qualitative study with an interpretive description design was conducted.^26,27^ This is an inductive and constructivist approach to developing knowledge to assist professionals in the applied health disciplines. Here, new insight is co-constructed iteratively between the researcher and data, going back and forth to understand complex clinical phenomena.^28^ It is specified by Hunt^28^ that the results of an interpretive description inquiry are organized into a coherent professional narrative to inform clinical practice.

### Recruitment and inclusion criteria

Informants were recruited based on purposeful sampling.^29^ To provide a broad and varied sample within a field with limited research, the following inclusion criteria were applied: (1) multidisciplinary healthcare professionals, (2) having clinical experience of municipality-based PPC, (3) working in primary healthcare, specialist healthcare, or non-governmental organizations (NGO).

The sample was recruited via a cancer coordinator in a municipality, and directly from a hospital-based PPC team and national NGOs, representing different municipalities and health regions. Sixteen professionals were approached, and all consented to participate. The informants were mainly female nurses, with a mean age of 47 years, with long clinical experience, and working in primary healthcare. The informants’ sociodemographic data is presented in Table 1.

**Table 1.** Informants’ sociodemographic variables (*N* = 16).

Variable	*N* (%)	Mean (SD)
Gender		
Female	14 (87.5)	
Male	2 (12.5)	
Age		47.4 (13.5)
Own children <18 years of age	7 (43.8)	
Profession		
Healthcare worker	1 (6.3)	
Registered nurse	12 (75)	
General practitioner	1 (6.3)	
Psychologist	1 (6.3)	
Other	1 (6.3)	
Years of work experience		24.9 (12.0)
Working in		
Primary healthcare	12 (75)	
Specialist healthcare	2 (12.5)	
NGO	2 (12.5)	

N, total number of informants; NGO, non-governmental organization (Child Cancer Organization); SD, standard deviations.

### Data collection

The data was collected through individual in-depth and semi-structured interviews by the third author. This approach was considered the most effective for data collection due to its flexibility in covering specific topics while allowing participants to introduce new insights. It enables researchers to deeply explore complex phenomena, ask clarifying follow-up questions, and capture a wide range of perspectives. After receiving the informants’ contact information, the interviewer contacted all informants by email, providing a presentation of the researcher, the aim and content of the project, a letter of information and consent, and scheduling time for the interviews. No relationship was established prior to study commencement. All interviews were conducted once, digitally and face-to-face on Zoom. Both the interviewer and informants were placed in a silent room at the workplace or at home, with no one else present. Before each interview, the researcher presented herself and repeated the aim, content, and the practical conduct of the interview. All interviews were based on a semi-structured interview guide with open-ended questions that were not pilot tested. The opening question was: “Can you please tell me about your experience of PPC in municipalities?” The informants were encouraged to talk about their experiences as freely as possible. Depending on the development of the interviews, follow-up questions revolved around important challenges and important factors in providing municipality-based PPC. The interviews lasted for between 45 and 75 min and were recorded digitally, but only the sound files were stored. No field notes were made during or after the interviews. The interviews were transcribed verbatim by the first author and an external research assistant. Following the transcription, the original audio recordings were securely disposed. To secure the informants’ confidentiality, names and other identifying characteristics were excluded. Data saturation, meaning that no new codes or themes emerged, was reached after 13 interviews. However, we decided to conduct interviews with all 16 recruited informants to ensure that data saturation was maintained and to obtain maximal variation.^26,30^ The transcribed interviews were not returned to the informants for a credibility check, as this process is time-consuming and could introduce contradictions during the analysis and knowledge development phases.^26^

### Data analysis

The transcribed data amounted to 132 pages of text and was analyzed using systematic text condensation (STC), which offers a reflexive and feasible process of analysis for qualitative data while maintaining responsible rigor.^31^ This approach aligns well with the study’s research question and design, providing a clear and systematic analysis process. It effectively identifies and extracts themes, breaking down data into manageable units to facilitate the recognition of patterns and relationships. Additionally, it preserves the context and meaning of the original data, thereby enhancing both transparency and reproducibility.^31^ STC consists of four stages: (1) total impression, (2) identifying and sorting meaning units, (3) condensation, and (4) synthesizing the findings. STC is also iterative, encouraging a back-and-forth process between the stages and involving several researchers to widen the analytic space, changing and adjusting the understanding of the data. The stepwise analyses process is elaborated in Table 2.

**Table 2.** The analyses process.

Step 1: Obtaining a total impression			
Process	Identified total impression		
The authors read the transcribed interviews individually and subsequently discussed their total impressions until consensus	Early involvement to build relationships, safety, and predictability Focusing on the whole family, including assessing their needs Dedicated, coordinated, competent team for PPC Close collaboration between various services in the municipality and specialist healthcare Building competence and confidence in the PPC team, and follow-up and guidance for healthcare professionals Need for service coordination and clarification of roles Acknowledging the need for bereavement support		
Step 2: Identifying meaning units			
Process	Identified meaning units		
The authors identified meaning units separately and marked them with a code. Codes were discussed until consensus, paying special attention to differences to widen the analytic space Associated text in all interviews was identified	Codes	Files	References
	Security and predictability (family and healthcare professionals)	16	178
	Coordination and multidisciplinary/interagency collaboration	16	151
	Healthcare professionals’ competence	16	124
	Information and communication	15	113
	Follow-up and cooperation with parents	15	102
	Early involvement: assessing, planning, preparedness	15	94
	Symptom management, procedures, and toolkit regarding the ill child	13	66
	Personal experience	16	65
	Guidance and debriefing for healthcare professionals	15	57
	Holistic family perspective	15	57
	Support for siblings	15	52
	Bereavement support	16	39
	Time of death	8	19
	*Files* *=* *(n) informants mentioning the meaning unit* *References* *=* *(n) mentions of the meaning unit*		
Step 3: Condensation from meaning units to themes			
Process	Identified themes		
The authors discussed the content of each code until consensus and condensed them into a bridging theme, elaborated by main themes and further by subthemes Nuances were explored, going back, and reorganizing/redefining themes where needed The findings were validated against the transcribed interviews	1 bridging theme 3 main themes 7 subthemes (see Table 3 for the results)		
Step 4: Synthesizing the findings			
Process	Findings		
The authors summarized the contents of the themes, identified quotes from the informants, and reevaluated	The themes were elaborated and illustrated by direct quotes		

In accordance with the first stage, all interviews were read, and all authors identified five or six initial themes individually before discussing them until consensus was achieved. In the second stage, the authors examined the material more thoroughly, identified different meaning units, and marked them with codes. Each researcher identified 8–13 codes, and through discussion, a consensus was reached on 13 codes. These were entered into the analysis program NVivo 14,^32^ and associated text in all interviews was identified. In the third stage, the content of each code was discussed and abstracted into a bridging theme, which was elaborated by three main themes and further by seven subthemes.

The findings were validated by comparison with the transcripts. In the fourth stage, the contents of the themes were summarized, nuances were explored, and quotes from the informants were identified. Throughout the process, the authors went back and forth, reorganizing the codes and abstraction of the content, exploring differing interpretations, and discussing until consensus was achieved.

Concerning preunderstanding, the first author had previously been involved in the larger project as a research assistant, while the second author was a novice in the field. These authors are female psychologists. The third author is a female professor with clinical experience in palliation and experienced in qualitative research. Throughout the analysis process, each author recognized and endeavored to bracket their preconceptions derived from their research field and personal experiences. This was done to ensure that the formulation of content was grounded in the informants’ articulated lifeworlds.^28^

### Ethical considerations

This study is part of a larger project researching important factors for municipality-based PPC from different perspectives. The study was considered by the Regional Committee for Ethics in Medical and Health Research (REK, reference number 435941), which concluded it to be outside the Norwegian Act on Medical and Health Research.^33^ The study was approved by the Norwegian Agency for Shared Services in Education and Research (SIKT ID: 270888) and implemented in the University of Bergen’s system for risk and compliance in research projects (RETTE, ID: R2082). The informants received written and verbal information about the study, including that participation was voluntary and that they could withdraw at any time. All provided their consent for participation, written or verbally on tape. The study was conducted according to the Declaration of Helsinki.^34^ The data was digitally stored in adherence with the University of Bergen’s guidelines. Consolidated criteria for reporting qualitative research (COREQ) was followed in reporting this study (Supplemental Material).^35^

## Results

From the analysis, the bridging theme “Establishing a sense of security and predictability for family and healthcare professionals” emerged as paramount for municipality-based PPC. The informants underlined that healthcare professionals’ confidence in their role was a prerequisite for the family to trust and adapt to municipality-based services. A condition for both was reciprocal trust and routine collaboration on a systemic level.

One of the most important things is that we in the team are confident enough in our work. Secure enough to do what we are supposed to do, so the sense of security is relayed to parents, other relatives, children, so they see that we know what we’re doing. (Informant 7)

This bridging theme was elaborated into three main themes related to how security and predictability could be achieved in different ways and on different levels. The main themes were further expanded into several subthemes, as outlined in Table 3 and described below.

**Table 3.** The study’s results.

*Bridging theme*: Establishing a sense of security and predictability for family and healthcare professionals	
Main themes	Subthemes
A comprehensive approach to the family	Optimizing the child’s quality of life Cooperation and care for parents through a crisis
	Being attentive toward siblings
Establishing and maintaining a dedicated, multidisciplinary pediatric palliative team in the municipality	Specialized pediatric palliative expertise in the team
	Professional guidance and support for the team
Collaboration and communication between involved services	Early referral to the municipality pediatric palliative team Coordination of services

### A comprehensive approach to the family

The first main theme highlighted a comprehensive approach to the whole family as an essential factor in municipality-based PPC. “*To take care of the whole complexity of the family’s needs, that is, and especially the practical, economic, material aspects*” (Informant 8). The participants acknowledged that this approach entailed the entire illness trajectory from diagnosis to follow-up of the family after bereavement. This main theme was further elaborated by three subthemes.

#### Optimizing the child’s quality of life

The first subtheme underlined that the main task for the healthcare professionals in the municipality was focusing on the quality of life for the ill child. This involved medical treatment and other crucial aspects of their well-being, such as social, psychological, and existential aspects, to promote a fulfilling life. “*You can’t really do anything to make them healthy again, but you can do something that contributes to making the remaining life they are going to live, and the death (. . .), as good as possible*” (Informant 12). Informants stated that healthcare professionals getting to know the child was a prerequisite for optimal care. This included knowledge about their condition, preferences, and interests, how to calm them, and how to adapt communication to their developmental stage. Some informants underlined the importance of a consistent healthcare team throughout the illness trajectory to achieve this.

Further, most informants emphasized the importance of the child being at home as much as possible and therefore regarded it as key that the municipality offered municipality-based care. This was considered crucial in fostering a sense of normality and safety, enabling activities that met the child’s social and psychological needs. For municipality healthcare professionals to provide this care, the informants underlined that a detailed medical plan had to be prepared, and all necessary equipment had to be ready in the home before the child was transferred from the hospital.

Additionally, the informants expressed different views regarding how much information the child should receive about their palliative situation. Most informants thought it better to be open with the child, although in cooperation with the parents. Some underlined how ethically difficult situations could arise when parents did not agree that the child should be informed and that healthcare professionals should be prepared to handle this dilemma.

#### Cooperation and care for parents through a crisis

The second subtheme emerged from the informants’ focus on the cooperation with and the care for the child’s primary caregivers as an important factor for municipality-based PPC. This entailed healthcare professionals engaging with the parents, both as allies in providing care for the child and as family members in a critical situation requiring support and help themselves. Informants stressed that involving parents in all decisions concerning the family promoted tailored and consistent care throughout the illness trajectory. One of the main areas for cooperation was in communicating with the child.

I think that as parents they know their child best. It’s very important that we are on the same team. What do you see now? What are you thinking now? That we involve them a lot, but also contribute with, if I see that it’s not right. . . Daring to present it in a good way, but that’s naturally very difficult. (Informant 1)

Several informants also mentioned acknowledging and addressing that the parents themselves were in a crisis as an important albeit challenging part of PPC. They recalled impediments to cooperation, ranging from parents being absent or omnipresent during healthcare professionals’ presence, having overly high expectations of or rejecting certain professionals, expressing anger or sadness, being debilitated by stress, exhaustion or family conflicts, or even being suicidal. “*We see that some parents withdraw from the child, instead of drawing closer to them (. . .). It comes down to this with working with. . . understanding what it’s like to work with people in a crisis*” (Informant 16).

In addition to cooperating with, and caring for, parents in a crisis, the informants saw a challenge arise in balancing these two, especially when they were contradictory—for example, if parents struggled to accept that the child had a life-threatening condition. Parents’ needs could oscillate between wanting control over procedures or logistics and needing respite to care for themselves. All informants stressed the importance of building a solid relationship with parents over time to prevent and handle these challenges.

Lastly, informants saw comprehensive care as entailing follow-up after the child’s passing, but with differing views of municipality healthcare professionals’ roles. Some thought they should be responsible for parental bereavement support, while others thought they should guide them to other available resources.

#### Being attentive toward siblings

The third subtheme reflected how healthcare professionals being attentive toward siblings was part of the comprehensive approach to the family. By the municipality providing municipality-based PPC, the informants experienced already being helpful to siblings by promoting normalcy, facilitating their engagement in regular activities, and reducing the feeling of being left out of the family. Apart from that, the participants described that support for siblings could unfold in several ways, such as directly helping (e.g., by talking with them about their challenges and, including them in care tasks), advising their parents, and establishing connections with the school and the family’s broader network. To identify the siblings’ needs, the importance of a trusting relationship was underlined.

Maybe try to get to know them a little, which interests and, like, build a sort of trust (. . .). That they maybe will tell you about something they are thinking of, (. . .) maybe they need something (. . .) to be able to handle the situation in their own way. (Informant 6)

Furthermore, a trusting relationship between municipality healthcare professionals and parents was thought to promote siblings’ well-being. Some informants experienced that parents in a crisis sometimes had limited capacity to care for siblings, which left healthcare professionals with more responsibility. Thus, confidence in healthcare professionals’ ability to independently care for the sick child empowered parents to dedicate time to their other children. Regarding the need for bereavement support, some of the informants emphasized the need for siblings to be offered professional help individually over time.

### Establishing and maintaining a dedicated, multidisciplinary pediatric palliative team in the municipality

The second main theme described the fact that the informants found establishing and maintaining a dedicated, multidisciplinary PPC team in municipalities as an important way to ensure the necessary PPC expertise.

(. . .) that [parents] see that we can take care of them in their homes, in the best possible way. And it gives them a sense of security (. . .) that the municipality has a team, and we can take care of them at home. (Informant 5)

For the team to function and provide comprehensive care over time, it emerged as crucial that the professionals receive expertise enhancement as well as routine emotional support, which was further elaborated by two subthemes.

#### Specialized multidisciplinary pediatric palliative expertise in the team

This first subtheme underscored how all informants emphasized the necessity of multidisciplinary and specialized expertise in municipality PPC. Organizing primary healthcare professionals into a dedicated team was described as an important way of ensuring expertise over time. The informants described that holistic and multidisciplinary expertise was required to meet the family’s various needs. This ranged from knowledge about child development, and symptom assessment and management, to familiarity with other public services such as the Norwegian Labor and Welfare Administration. Skills related to building connections with the family were emphasized, especially communication skills both concerning parents and children, including communicating with children not being able to communicate verbally: “*You will always learn the procedures (. . .), but it is how you meet the parents that is important. How you communicate with the children and parents*” (Informant 1). Moreover, knowledge about normal reactions to crises was highlighted as being imperative for healthcare professionals to trust their ability to help the family cope with these reactions.

In addition to a solid foundation of skills regarding PPC, informants stated the need for this expertise to be actively maintained, especially because pediatric palliative cases seldom occur. Several ways to achieve this were proposed, such as shadowing the pediatric palliation specialists at the hospital and internal courses.

#### Professional guidance and support for the team

The second subtheme reflected the involved healthcare professionals’ need for professional guidance and emotional support throughout the illness trajectory. Many informants expressed uncertainty regarding their ability to cope professionally with the emotional aspects of PPC, and several had experienced being part of a child’s palliative treatment as emotionally taxing. Additionally, several informants had children themselves and emphasized how this intensified their emotional reactions, which made them more challenging to navigate. However, being a parent themselves was also highlighted as a potential strength, as it provided a level of understanding and empathy that is essential for PPC. In summary, informants stressed the need to find a balance between taking care of themselves and taking care of the family. To achieve this, several considerations were identified.

First, it emerged as paramount that the team was experienced as a safe and secure entity. Therefore, providing the team with professional guidance from a psychologist, as well as support from the team leader and each other, was outlined as important. Most informants stated how this provision of support should be a priority—both regularly and after difficult situations.

It’s going to be quite tough, but then we must rely on each other. Because you must, in a way. . . you can’t go home without talking about it. Otherwise, it will fall apart for you one day. (. . .) Talk about it. It helps me a lot. Talk it through. (Informant 2)

Moreover, the importance of the team leader attending these sessions was underscored by several informants, as this increased the opportunity for leaders to provide individual and practical support if necessary. This was also the case after the death of the child, as this could evoke strong reactions in the healthcare professionals. Many highlighted the significance of being allowed to see the process through—for example by attending the child’s funeral.

### Collaboration and communication between involved services

The third main theme emerging from the analysis was centered on the need for collaboration and communication between the municipality services and across systemic levels involved in providing municipality-based PPC. Some informants based this need on negative experiences:

I was in some of those meetings, like, on a systemic level in the municipality. (. . .) I experienced that there was randomness. There was uncertainty about who should do what. (. . .) It was, like, not a seamless and, like, predictable course for the family and the parents on who should do what. (Informant 8)

Routines for integrating the different services were seen by the informants as a prerequisite for building a safe and predictable illness trajectory for the patient, the family, and the professionals providing the care. This third main theme was elaborated by two subthemes.

#### Early referral to the municipality pediatric palliative team

The first subtheme reflected that the informants thought it essential for the municipality healthcare team to be involved early in the child’s illness trajectory, based on earlier experiences of being included too late. Some informants thought it advantageous to inform the municipality pediatric palliative team about the family at the time of diagnosis. Being able to meet the family during a less critical phase of the illness trajectory was reported to promote a trusting relationship: “*In my experience, the earlier we come in [to the case], the better we can build relationships and contribute to a sense of security*” (Informant 11). Moreover, early referrals ensured having time to prepare for the transition to municipality-based care—examining the family’s needs and connecting relevant services and logistics, such as equipment, training, and staff reorganization. Finally, the participants stressed the importance of extending the overlap between the PPC team in the municipality and the hospital. Ensuring that the family was supported over time by well-integrated and prepared public services was thought to improve continuity of care, thereby providing them with a greater sense of security and predictability.

#### Coordination of services

The second subtheme reflected the informants’ emphasis on the need for coordination of the services involved in municipality-based PPC, clarifying the different roles and responsibilities before and during specific cases. Several informants recommended having a designated coordinator in the municipality to oversee and facilitate regular meetings, continuous dialogue on current issues, and collaborative plans for treatment and emergencies. Some specified that the coordinator should be the family’s primary contact, to ease the logistical burden on parents and promote a predictable process.

Across all the services involved, the importance of a constructive relationship between the hospital and the municipality team was highlighted. Some informants recalled how both the hospital and the municipality team could be hesitant toward transferring care, and that both parties should strive to be more unified in the support of the family to establish the necessary trust for a seamless transition to home care.

I have experiences where [the hospital] owns [the patients] for too long. And that parents are very skeptical of someone else coming into the picture if they have received a lot (. . .) of follow-up from [the hospital]. (Informant 11)

The need for the family’s general practitioner to be part of the collaboration between the hospital specialists and the municipality team was accentuated, especially to prevent uncertainty or contradictory messages to the healthcare professionals providing care in the home. Moreover, the existence of clear policies on who they could contact when in need of more specialized competence was stated as central to providing safe and predictable care at home. Furthermore, informants requested routines for interdisciplinary debriefs with all involved services after the child had died, to review the formal collaboration and treatment. This was thought to strengthen continuity and preparedness for future cases. Some informants mentioned that achieving and maintaining municipality-based PPC required management commitment in all the involved instances, especially in the communities’ administrative and political management.

## Discussion

This study explored what healthcare professionals experience as important factors in providing municipality-based PPC. An overarching finding was the necessity of ensuring security and predictability for both families and healthcare professionals, serving as a prerequisite for effective PPC in primary healthcare settings. To achieve this, several factors across different levels were identified as essential.

First, our findings acknowledge the complexity of PPC, which includes diversity in diagnosis, varying developmental stages,^4^ and limited exposure to children in palliative care within municipalities.^20^ These factors place high demands on healthcare professionals’ level of competence^13,21,23,24^ and emotional coping abilities.^21–23,36,37^ However, regarding the goal of optimizing the child’s quality of life,^3,4,7^ our results support the provision of municipality-based PPC. Being at home fosters a sense of normalcy and provides better opportunities to address the child’s social, psychological, and existential needs. When a child faces serious illness, the entire family is impacted,^4^ and our results underscore the importance of holistic and multidisciplinary support for the entire family, also aligning with current guidelines.^3,7^ Nevertheless, our findings stress that a holistic approach does not occur automatically by providing municipality-based PPC. Healthcare professionals in the municipality are typically accustomed to providing patient-centered care for adults, which makes the transition to family-centered care for children challenging.^20^

In the Netherlands, an individualized PPC plan has recently been developed, covering physical, psychological, spiritual, and social functioning, and emphasizing patients’ and parents’ preferences and desires. This has been shown to improve care and ease the transition from hospital to home.^38^ Similar strategies for pediatric advanced care planning have also been found to be beneficial,^39^ whereby initiation of end-of-life discussions at an early stage of treatment stands out as crucial.^39–41^ This is consistent with our findings and underscores the necessity for assessing the family’s needs in the municipality at an early stage to enable tailored and predictable care, especially because of the unpredictable nature of PPC.

As previously documented,^25^ our findings emphasize that parents are key partners in caring for the child. The importance of establishing a trusting relationship with the family was further underscored, which is consistent with previous studies.^12,14,23^ However, our study adds to this element by highlighting the difficulties faced by municipality healthcare professionals in building such relationships, as managing cooperation with parents in crisis emerged as a significant challenge. Moreover, studies on parents’ experiences have found that they lack trust in municipality healthcare professionals^11^ and can feel misunderstood.^14^ Consequently, this lack of trust may jeopardize the overall sense of security and lead parents to prefer hospital care. Thus, healthcare professionals’ capability to recognize and manage parental crisis reactions emerges as a crucial factor in providing municipality-based PPC.

Healthcare professionals in Norway are legally obligated to care for siblings of seriously ill children.^10^ However, studies suggest that siblings still risk being deprioritized.^11,42^ Our findings highlight that healthcare providers recognize this challenge and want to be more attentive and supportive of siblings when providing care at home. Yet, expecting healthcare professionals to deliver daily care to shoulder this responsibility alone, amidst numerous competing demands, may be unreasonable. This underscores the need for a systemic solution. A recent meta-analysis^42^ proposed coordinated efforts among hospitals, municipality health services, and school nurses, which our findings support. Furthermore, this approach may serve as a preventive strategy that supports siblings’ coping and reduces the workload of healthcare professionals in the palliative care team.

The findings further suggest that healthcare professionals may not fully recognize the importance of providing bereavement support to families after the death of the child, with varying views on what this support should entail and who should be responsible for it. This echoes previous findings that suggest deficiencies in municipal support for bereaved families,^11^ even though losing a child is recognized as one of the most distressing events one can experience,^43^ often accompanied by serious grief reactions that impair functioning over time.^44,45^ These findings emphasize the necessity for clearer guidelines in this area.

As prior research has indicated, our findings emphasize the challenge of establishing and maintaining sufficient expertise among healthcare professionals in the municipality—partly due to the complexity of PPC and the limited number of cases.^20,23,46^ Two recent Norwegian studies have shown parental dissatisfaction with the level of expertise in municipalities,^11,14^ which may lead parents to mainly rely on hospital-based care, consequently spending less time at home than originally preferred. Additionally, inadequate expertise may lead parents to take on more caregiving responsibilities, potentially compromising not only their own quality of life but also that of the child and siblings. Our findings suggest the establishment of dedicated and multidisciplinary teams in the municipality as a possible solution, which resonates with national and international guidelines, as well as previous findings.^5,7,24^ To ensure professional well-being for healthcare professionals in such teams, our results emphasize the need for sufficient training, a strong sense of trust among team members, professional guidance, emotional support, and routines for debriefing, both within the team and individually. This is in line with existing literature.^21–23,36^ Furthermore, the opportunity for healthcare professionals to see the process through was highlighted—for example, by attending the child’s funeral, as also found in a previous study.^22^ Overall challenges persist in implementing PPC teams in municipalities, including the dependence on local resources and priorities. Collaboration between municipalities could therefore be considered for small municipalities, as well as participating in regional networks for PPC, as suggested by governmental guidelines.^47^

A central finding was the importance of systemic collaboration, especially between the hospital and the municipality healthcare services, to enhance predictability and seamless trajectories for both the family and the involved healthcare professionals. This is supported by previous studies^20,39^ and guidelines.^4,7^ Further, building upon previous findings,^11,20,23,25^ our results underscore the importance of early referrals from specialists to municipality services. Early referral seems to be crucial for the transferring of knowledge and seamless collaboration in the child’s treatment. Additionally, early involvement of municipality healthcare professionals was believed to enhance parental trust and a sense of security. Several informants recalled negative experiences, such as hurried preparations when becoming involved at a late stage in the child’s illness, which resulted in limited assessment of the family’s holistic needs and compromised professional training in the municipality. This may hinder the establishment of trust between the family and the municipality. Our results further contribute to expanding this knowledge by suggesting that late referrals may stem from uncertainty in specialist services regarding when and to whom they should refer families. Concerns about the municipality’s readiness in terms of expertise and routines for PPC may also play a role. Remarkably, this hesitation appears to be mutual, as municipality healthcare professionals also expressed uncertainty about their capacity to deliver adequate municipality-based PPC. One might speculate as to whether this could result in a self-fulfilling prophecy: late referrals make it impossible for the municipality to adequately map the family’s needs and sufficiently prepare for the transition to municipality-based care, which reinforces the assumption that they may not be able to provide adequate care. Clinical implications thus include ensuring clear routines for early referrals. Future research should address this surprising mutual uncertainty.

Our findings further indicate that the clarification of responsibilities across the services is essential. To ensure collaboration as intended, our results suggest having a designated care coordinator in the municipality, consistent with previous recommendations.^11,13,25^ This appears to be crucial for coordinating municipality services, facilitating interdisciplinary meetings, and communicating with the specialist healthcare service and general practitioner, thereby relieving parents of this responsibility. Finally, our findings emphasize the demand for integrated service planning at management levels, including that municipality and specialist services receive concordant training and routines to facilitate collaboration. Management commitment and attention to PPC in the municipality over time seem necessary to implement realistic routines, educate healthcare professionals in PPC, maintain contact with other services, and ensure continuous evaluation.

### Strengths and limitations

This study is centered on the exploration of healthcare professionals’ experiences, highlighting the critical elements that contribute to the efficacy of home-based PPC administered by municipal services. The significance of this research is underscored by the scarcity of existing studies examining this viewpoint. Further strengths of this study include the qualitative approach, which amplifies the voices of experienced and multidisciplinary healthcare professionals. Their diverse experiences and backgrounds across various levels of healthcare contribute to a comprehensive dataset, and the achievement of data saturation further strengthens the findings. Furthermore, the authors’ varied experience in PPC and professional background, strengthens the study by bringing diverse perspectives to the interpretative discussions. However, having an even more diverse sample in terms of discipline could have enriched the study. The qualitative method and the conducting of the study in the Norwegian healthcare system may limit the transferability of findings to other contexts. Nevertheless, a high level of intercoder agreement, transparent analysis, and consistent findings across informants, as well as alignment with previous research, strengthen the findings.

## Conclusion

This study has identified important and previously underexplored factors for municipality-based PPC from healthcare professionals’ perspective. Common among them is their role in ensuring security and predictability for the family and healthcare professionals. The value of a holistic, family-centered approach to municipality-based PPC in the municipality is noted, including optimizing the child’s quality of life and supporting parents and siblings. Municipality-based PPC is complex, and to ensure sufficient competence for healthcare professionals, establishing dedicated, multidisciplinary teams is proposed. The need for emotional support and professional guidance for team members is highlighted. Clear routines for early referrals from specialists to municipality healthcare, clear routines for municipality-based PPC trajectories, a designated coordinator in the municipality, and sufficient resources are recommended. Moving forward, further research is needed to better understand how municipality-based PPC in municipalities can meet the needs of both families and healthcare professionals.

## Supplemental Material

sj-docx-1-pcr-10.1177_26323524241312915 – Supplemental material for Important factors in municipality-based pediatric palliation from healthcare professionals’ perspective: A qualitative study Supplemental material, sj-docx-1-pcr-10.1177_26323524241312915 for Important factors in municipality-based pediatric palliation from healthcare professionals’ perspective: A qualitative study by Kristin Skjærseth, Natalie Preminger and May Aasebø Hauken in Palliative Care and Social Practice

## Declarations

**Ethics approval and consent to participate:** This study is part of a larger project related to municipality-based pediatric palliative care. The study was considered by the Regional Committee for Ethics in Medical and Health Research (REK, reference number 435941) which concluded it to be outside the Norwegian Act on Medical and Health Research. The study was approved by the Norwegian Agency for Shared Services in Education and Research (SIKT, reference number 270888) and implemented in the University of Bergen’s system for risk and compliance in research projects (RETTE ID: R2082). The informants received written and verbal information about the study, including that participation was voluntary and that they could withdraw at any time.

**Consent for publication:** All informants received written and verbal information about the publication of data, whereby all consented.

**Availability of data and materials:** The data that support the findings of this study are not publicly available due to privacy or ethical restrictions. The data are available on reasonable request from the third author.
